# Transfer Learning in Breast Cancer Diagnoses via Ultrasound Imaging

**DOI:** 10.3390/cancers13040738

**Published:** 2021-02-10

**Authors:** Gelan Ayana, Kokeb Dese, Se-woon Choe

**Affiliations:** 1 Department of Medical IT Convergence Engineering, Kumoh National Institute of Technology, Gumi 39253, Korea; gelan@kumoh.ac.kr; 2 School of Biomedical Engineering, Jimma University, Jimma 378, Ethiopia; kokebdese86@gmail.com; 3 Department of IT Convergence Engineering, Kumoh National Institute of Technology, Gumi 39253, Korea

**Keywords:** transfer learning, breast cancer, ultrasound

## Abstract

**Simple Summary:**

Transfer learning plays a major role in medical image analyses; however, obtaining adequate training image datasets for machine learning algorithms can be challenging. Although many studies have attempted to employ transfer learning in medical image analyses, thus far, only a few review articles regarding the application of transfer learning to medical image analyses have been published. Moreover, reviews on the application of transfer learning in ultrasound breast imaging are rare. This work reviews previous studies that focused on detecting breast cancer from ultrasound images by using transfer learning, in order to summarize existing methods and identify their advantages and shortcomings. Additionally, this review presents potential future research directions for applying transfer learning in ultrasound imaging for the purposes of breast cancer detection and diagnoses. This review is expected to be significantly helpful in guiding researchers to identify potential improved methods and areas that can be improved through further research on transfer learning-based ultrasound breast imaging.

**Abstract:**

Transfer learning is a machine learning approach that reuses a learning method developed for a task as the starting point for a model on a target task. The goal of transfer learning is to improve performance of target learners by transferring the knowledge contained in other (but related) source domains. As a result, the need for large numbers of target-domain data is lowered for constructing target learners. Due to this immense property, transfer learning techniques are frequently used in ultrasound breast cancer image analyses. In this review, we focus on transfer learning methods applied on ultrasound breast image classification and detection from the perspective of transfer learning approaches, pre-processing, pre-training models, and convolutional neural network (CNN) models. Finally, comparison of different works is carried out, and challenges—as well as outlooks—are discussed.

## 1. Introduction

Breast cancer is the second leading cause of death in women; 12.5% of women from different societies worldwide are diagnosed with breast cancer [1]. According to previous studies, early detection of breast cancer is crucial because it can contribute to up to a 40% decrease in mortality rate [2,3]. Currently, the ultrasound imaging technique has emerged as a popular imaging modality for the diagnoses of breast cancer, especially in young women with dense breasts [4]. This is because ultrasound (US) imaging is a non-invasive procedure and it can efficiently capture tissue properties [5,6,7]. Studies have shown that the false negative recognition rate in other breast diagnosis methods, such as biopsy and mammography (MG), decreased on using different modalities, such as US imaging [2]. Additionally, ultrasound imaging methods can be used to improve the tumor detection rate by up to 17% during breast cancer diagnoses [6]. Furthermore, the number of non-essential biopsies can be decreased by approximately 40%, thereby reducing medication costs [5]. An additional benefit of ultrasound imaging is that it uses non-ionizing radiation, which does not negatively affect health and requires relatively simple technology [7]. Therefore, ultrasound scanners are cheaper and more portable than mammography [5,6,7,8]. However, ultrasonic systems are not a standalone modality for breast cancer diagnoses [6,7]; instead, they are integrated with mammography and histological observations to validate results [8]. To improve the diagnostic capacity of ultrasound imaging, several studies have employed existing technologies [9]. Machine learning has solved many of the problems associated with ultrasound in terms of the classification, detection, and segmentation of breast cancer, such as false positive rates, limitation in indicating changes caused by cancer, lower applicability for treatment monitoring, and subjective judgments [10,11,12]. However, many machine learning methods perform well only under a common assumption, i.e., the training and test data are obtained from the same feature space and have the same distribution [13]. When the distribution changes, most numerical values of the models need to be constructed from scratch using newly collected training data [11,12,13]. In medical applications, including breast ultrasound imaging, it is difficult to collect the required training data and construct models in this manner [14]. Thus, it is advisable to minimize the need and effort required for acquiring the training data [13,14]. In such scenarios, transfer learning from one task to the target task would be desirable [15]. Transfer learning enables the use of a model previously trained on another domain as the target for learning [16]. Thus, it reduces the need and effort required to collect additional training data for learning [10,11,12,13,14,15,16].

Transfer learning is based on the principle that previously learned knowledge can be exceptionally implemented to solve new problems in a more efficient and effective manner [17,18]. Thus, transfer learning requires established machine learning approaches that retain and reuse previously learned knowledge [19,20,21]. Transfer learning was recently applied to breast cancer imaging in 2016, following the emergence of several convolutional neural network (CNN) models, including AlexNet, VGGNet, GoogLeNet, ResNet, and Inception, to solve visual classification tasks in natural images that are trained on natural image database such as ImageNet [22]. The first application of transfer learning to breast cancer imaging was reported in 2016 by Hyunh et al., where they assessed the performance achieved by using features transferred from pre-trained deep CNNs for classifying breast cancer through computer-aided diagnosis (CADx) [23]. Following this, Byra et al. published a paper where they proposed a neural transfer learning approach for breast lesion classification through ultrasound [24]. Shortly after this, Yap et al. [25] published their work, which proposed the use of deep neural learning methods for breast cancer detection; they studied three different methods—a patch-based LeNet approach, a U-Net model, and a transfer learning method—with a pre-trained fully convolutional network, AlexNet. Following these works, a large number of articles have been published in the area of applying transfer learning for breast ultrasound imaging [26,27,28,29].

This work reviews articles that focus on breast cancer imaging using transfer learning to summarize existing methods and identify their strengths and weaknesses. Further, it presents potential future research directions for transfer learning in breast cancer imaging using ultrasound. The review will be instrumental in guiding researchers to identify potential improved methods as well as areas that would benefit from future research on transfer learning-based ultrasound breast imaging.

## 2. Transfer Learning

### 2.1. Overview of Transfer Learning

**Figure 1:**
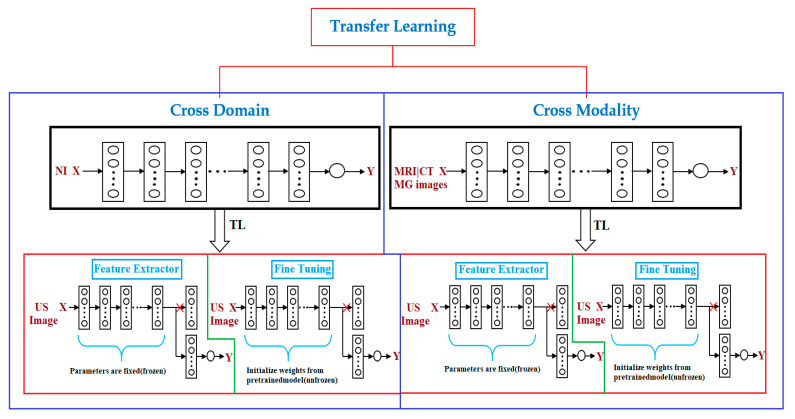
Transfer learning (TL) methods. There are two types of transfer learning used for breast cancer diagnosis via ultrasound imaging, depending on the source of pre-training data: cross-domain (model pre-trained on natural images is used) and cross-modal (model pre-trained on medical images is used). These two transfer learning approaches are feature extractors (convolution layers are used as a frozen feature extractor to match with a new task such as breast cancer classification) and fine-tuning (where instead of freezing convolution layers of the well-trained convolutional neural network (CNN) model, their weights are updated during the training process). X, input; Y, output; NI, natural image; MRI, magnetic resonance imaging; MG, mammography; CT, computed tomography; US, ultrasound.

Transfer learning is a popular approach for building machine learning models without concerns about the amount of available data [30]. Training a deep model may require a significant amount of data and computational resources; however, transfer learning can help address this issue. In many cases, a previously established model can be adapted to other problems [31] via transfer learning. For instance, it is possible to use a model that has been trained for one task, such as classifying cell types, and then fine-tuning it to accomplish another task, such as classifying tumors. Transfer learning is a particularly indispensable approach in tasks related to computer vision. Studies on transfer learning have shown [31,32,33] that features learned from significantly large image sets such as ImageNet are highly transferable to a variety of image recognition tasks. There are two approaches to transferring knowledge from one model to another. The popular approach is to change the last layer of the previously trained model and replace it with a randomly initialized one [34]. Following this, only the parameters in the top layer are trained for the new task, whereas all other parameters remain fixed. This method can be considered to be the application of the transferred model as a feature extractor [35], because the fixed portion acts as a feature extractor (Figure 1), while the top layer acts as a traditional, fully connected neural network layer without any special assumptions regarding the input [34,35]. This approach works better if the data and tasks are similar to the data and task on which the original model was trained. In cases where there is limited data to train a model for the target task, this type of transfer learning might be the only option to train a model without overfitting, because having fewer parameters to train also reduces the risk of overfitting [36]. In cases where more data is available for training, which is rare in medical settings, it is possible to unfreeze transferred parameters and train the entire network [34,35,36,37]. In this case, essentially, the initial values of the parameters are transferred [37]. The task of initializing the weights using a pre-trained model instead of initializing them randomly can provide the model with a favorable beginning and improve the rate of convergence [36,37] and fine-tuning. To preserve the initialization from pre-training, it is common practice to lower the learning rate by one order of magnitude [38,39]. To prevent changing the transferred parameters too early, it is customary to start with frozen parameters [40,41,42,43,44], train only randomly initialized layers until they converge, and then unfreeze all parameters and fine-tune (Figure 1) the entire network. Transfer learning is particularly useful when there is a limited amount of data for one task and a large volume of data for another similar task, or when there exists a model that has already been trained on such data [45]. However, even if there is sufficient data for training a model from scratch and the tasks are not related, initializing the parameters using a pre-trained model is still better than random initialization [46].

### 2.2. Advantages of Transfer Learning

The main advantages of transfer learning include reducing training time, providing better performance for neural networks, and requiring limited data [47,48,49,50]. In neural networks trained on a large set of images, the early layer parameters resemble each other regardless of the specific task they have been trained on [16,47]. For example, CNNs tend to learn edges, textures, and patterns in the first layers [31], and these layers capture the features that are broadly useful for analyzing the natural images [47]. Features that detect edges, corners, shapes, textures, and different types of illuminants can be considered as generic feature extractors and can be used in many different types of settings [30,31,32,33]. The closer we get to the output, the more specific features the layers tend to learn [48,49,50]. For example, the last layer in a network that has been trained for classification would be highly specific to that classification task [49]. If the model was trained to classify tumors, one unit would respond only to the images of a specific tumor [23,24,25,26,27,28]. Transferring all layers except the top layer is the most common type of transfer learning [17,18,19,20]. Generally, it is possible to transfer the first *n* layers from a pre-trained model to a target network and randomly initialize the rest [51]. Technically, the transferred part does not have to be the first layer; if the tasks are similar, the type of input data is slightly different [21]. It is also possible to transfer the last layers [33]. For example, consider a tumor recognition model that has been trained on gray scale images and that the target is to build a tumor recognition model that inputs images that are colored in addition to gray scale data. Given that significant amounts of the data are not available to train a new model from scratch, it may be effective to transfer the latter layers and re-train the early ones [52,53]. Therefore, transfer learning is useful in the case where there is insufficient data for a new domain that is to be handled by a neural network and there exists a large pre-existing data pool that can be transferred to a target problem [47,48,49,50,51,52,53]. Transfer learning facilitates the building of a solid machine learning model with comparatively smaller training data because the model is already trained [53]. This is especially valuable in medical image processing because most of the time, data annotating persons are required to create large labeled datasets [24,25,26,27,28,29]. Furthermore, training time is minimized because it can reduce the time required to train a new deep neural network from the beginning in the case of complex target task [48,49].

### 2.3. Transfer Learning Approaches

Transfer learning has enabled researchers in the field of medical imaging, where there is a scarcity of data, to address the issue of small sample datasets and achieve better performance [13]. Transfer learning can be divided into two types—cross-domain and cross-modal transfer learning—based on whether the target and source data belong to the same domain [54,55]. Cross-domain transfer learning is a popular method for achieving a range of tasks in medical ultrasound image analyses [9]. In machine learning, the pre-training of models is conventionally accomplished on large sample datasets, and large training data ensure outstanding performance; however, this is far from reality, making the approach unsuitable in the medical imaging domain [56]. In the case of small training samples, the domain-specific models trained from scratch can work better [57,58,59,60] relative to transfer learning from a neural network model that has been pre-trained with large training samples in another domain, such as the natural image database of ImageNet. One of the reasons for this is that the gauging from the unprocessed image to the feature vectors used for a particular task, such as classification in the medical case, is sophisticated in the pre-trained case and requires a large training sample for improved generalization [58,59,60]. Instead, an exclusively designed small network will be ideal for limited training datasets that are usually experienced in medical imaging [13,58,59]. Furthermore, models trained on natural images are not suitable for medical images because medical images typically have low contrast and rich textures [61,62]. In such cases, cross-modal transfer learning performs better than cross-domain transfer learning [63]. In medical cases, especially in breast imaging, different modalities, such as magnetic resonance imaging (MRI), mammography (MG), computed tomography (CT), and ultrasound (US) are frequently used in the diagnostic workflow [63,64,65]. Mammography (i.e., X-ray) and ultrasound are the first-line screening methods for breast cancer examination, and it is trivial to collect large training samples compared to MRI and CT [66,67,68]. Breast MRI is a more costly, time-consuming method, and it is commonly used for screening high-risk populations, making it considerably difficult to acquire datasets and ground-truth annotation in the case of MRIs, as compared to ultrasound and mammograms [29]. In such instances, cross-modal transfer learning is an optimal approach [69,70]. A few experiments [29] have demonstrated the superiority of cross-modal transfer learning over cross-domain transfer learning for a given task in the case of smaller training datasets.

There are two popular approaches for transfer learning: feature extraction and fine-tuning [71] (Figure 1).

#### 2.3.1. Feature Extracting

The feature extracting approach harnesses a well-trained CNN model on a large dataset such as ImageNet, which makes it a feature extractor for the new target domain, for instance, breast ultrasound imaging [72]. Particularly, all convolution layers of the well-trained CNN model are fixed, whereas the fully connected layers are cleared up [31,32,33,34,35,36,37,38,39]. The convolution layers are used as a frozen feature extractor to match with a new task, such as a breast cancer classification task [41,42,43,44,45]. The extracted features are then supplied to a classifier that can form fully connected layers [45]. Lastly, the new classifier is only trained throughout the training process instead of the entire network [51,52,53].

#### 2.3.2. Fine-Tuning

A fine-tuning approach, such as that of the feature extractor, utilizes a well-trained CNN model on a large dataset, such as ImageNet, as the base and supersedes the CNN layers with new CNN layers [73,74]. In fine-tuning, instead of freezing the convolution layers of the well-trained CNN model, their weights are updated during the training process [51,52,53]. This is implemented by initializing the weights of the convolution layers of a new model with the pre-trained weights of the already well-trained CNN model, and initializing the classifier layers with arbitrary random weights. In fine-tuning, the entire network is trained during the training process [41,42,43,44,45].

#### 2.3.3. Feature Extracting vs. Fine-Tuning

Two transfer learning strategies were identified: feature extractor and fine-tuning. The feature extractor has the additional benefit of not requiring the training of a neural network, allowing the extracted features to be easily plugged into existing image analysis procedures [72]. Both these strategies are popular and have been widely applied [75,76]. However, a few authors have performed an intensive investigation to determine the strategy that yields the best results. In [24], three training approaches are proposed: a CNN architecture trained from scratch, a transfer learning approach with a pre-trained VGG16 CNN architecture further trained on ultrasound images, and a fine-tuned learning approach where the deep learning parameters are fine-tuned. The experimental results from [24] demonstrated that the fine-tuned model had the best performance (accuracy = 0.97, area under curve (AUC) = 0.98), with pre-training on ultrasound images. In [24] and [26], both the feature extraction (AUC = 0.849) and fine-tuning (AUC = 0.895) approaches were used, and the fine-tuning approach exhibited better performance. These results justify the fact that almost all of the previous studies on transfer learning applied to breast ultrasound [24,25,26,27,28,29] used fine-tuning to achieve superior performance (AUC = 0.895). However, in the performance analysis, the above conclusion does not provide sufficient insights into drawing a clear conclusion, because different studies used different methods (see Section 2.5) in terms of pre-processing, which highly affected performance; others even used different performance analysis metrics [23,24,25,26,27,28,29].

### 2.4. Pre-Training Model and Dataset

The most common pre-training models used for transfer learning in breast ultrasound are the VGG19, VGG16, AlexNet, and InceptionV3 models; VGG is the most common, followed by AlexNet and Inception, which are the least common. A comparison of the different pre-training models is not useful to determine the pre-training model that is better than the others for transfer learning in breast ultrasound [23,24,25,26,27,28,29]. However, one study [26], showed that Inception V3 outperforms VGG19, where the authors evaluated the impact of the ultrasound image reconstruction method on breast lesion classification using a neural transfer learning. In their study, a better overall classification performance was obtained for the classifier with the pre-training model using InceptionV3, which exhibited an AUC of 0.857. In the case of the VGG19 neural network, the AUC was 0.822.

Dataset usage for the pre-training of breast ultrasound transfer learning methods depends on whether cross-domain or cross-modal transfer learning methods are implemented [57,58,59,60]. In the case of cross-domain transfer learning, natural image datasets, such as ImageNet, are utilized as a pre-training dataset, whereas in the case of cross-modal transfer learning, datasets of MRI, CT, or MG images are utilized for pre-training the CNNs [23,24,25,26,27,28,29]. In the latter case, most researchers used their own data, although some used publicly available datasets. In breast ultrasound transfer learning, ImageNet is used, in most cases, as a pre-training dataset [23,24,25,26,27,28,29].

- ImageNet: ImageNet is a large image database designed for use in image recognition [77,78,79]. It comprise more than 14 million images that have been hand-annotated to indicate the pictured objects. ImageNet is categorized into more than 20,000 categories with a typical category consisting of several images. The third-party image URLs repository of annotations is freely accessible directly from ImageNet, although ImageNet does not own the images.

### 2.5. Pre-Processing

The pre-processing required for applying transfer learning to breast ultrasound accomplishes two objectives [24,26]. The first is to compress the dynamic range of ultrasound signals to fit on the screen directly, and the second is to enlarge the dataset and reduce class imbalance. To achieve the first objective, [26] used a common method for ultrasound image analysis. First, the envelope of each raw ultrasound signal was calculated using the Hilbert transform. Next, the envelope was log-compressed, a specific threshold level was selected, and the log-compressed amplitude was mapped to the range of [0, 255]. In [24], Byra et.al used a matching layer where they proposed adjusting the grayscale ultrasound images to the pre-trained convolution neural network model instead of replicating grayscale images through the channels or changing the lower convolution layer of the CNN. Augmentation is used to achieve the second objective, which involves enlarging the dataset. Enlarging the amount of labeled data generally enhances the performance of CNN models [24,26]. Data augmentation is the process of synthetic data generation for training by producing variations in the original dataset [80,81]. For image data, the augmentation process involves different image manipulation techniques, such as rotation, translation, scaling, and flipping arrangements [81]. The challenging part for data augmentation are memory and computational constraints [82]. There are two popular data augmentation methods: online and offline data augmentation [83]. Online data augmentation is carried out on the fly during training, whereas offline data augmentation produces data in advance and stores it in memory [83]. The online approach saves storage but results in a longer training time, whereas the offline approach is faster in terms of training, although it consumes a large amount of memory [80,81,82,83].

### 2.6. Convolutional Neural Network

A CNN is a feed-forward neural network commonly used in ultrasound breast cancer image analysis [84]. The main advantage of the CNN is its accuracy in image recognition; however, it involves a high computational cost and requires numerous training data [85]. A CNN generally comprises an input layer, one or many convolution layers, pooling layers, and a fully connected layer [74]. The following are the most commonly used CNN models used for transfer learning with breast ultrasound images [84].

- AlexNet: the AlexNet architecture is composed of eight layers. The first layers of AlexNet are the convolutional layers, and the next layer is a max-pooling layer for data dimension reduction [77,78,79]. AlexNet uses a rectified linear unit (ReLU) for the activation function, which offers faster training than other activation functions. The remaining three layers are the fully connected layers.
- VGGNet: VGG16 was the first CNN introduced by the Visual Geometry Group (VGG); this was followed by VGG19; VGG16 and VGG19 becoming two excellent architectures on ImageNet [85]. VGGNet models afford better performance than AlexNet by superseding large kernel-sized filters with various small kernel-sized filters; thus, VGG16 and VGG19 comprise 13 and 16 convolution layers, respectively [84,85,86].
- Inception: this is a GoogLeNet model focused on improving the efficiency of VGGNet from the perspective of memory usage and runtime without reducing performance accuracy [86,87,88,89]. To achieve this, it removes the activation functions of VGGNet that are iterative or zero [86]. Therefore, GoogLeNet came up with and added a module known as Inception, which approximates scattered connections between the activation functions [87]. Following InceptionV1, the architecture was improved in three subsequent versions [88,89]. InceptionV2 used batch normalization for training, and InceptionV3 introduced the factorization method to enhance the computational complexity of convolution layers. InceptionV4 brought about a similar comprehensive type of Inception-V3 architecture with a larger number of inception modules [89].

## 3. Results

To identify the relevant studies, main databases were searched, including Google scholars, PubMed, MEDLINE, IEEE, and others, as well as conference proceedings such as Medical Image Computing and Computer Assisted Intervention (MICCAI), Society of Photo-Optical Instrumentation Engineers (SPIE), Engineering in Medicine and Biology Society (EMBC), IEEE International Symposium on Biomedical Imaging (ISBI), and others, for articles published until September 2020. The keywords used for searching in this review were: “Transfer Learning” AND “Breast Images” OR “Breast Cancer Image” OR “Breast Cancer Classification” OR “Breast Cancer Diagnoses”. We identified 34 potentially relevant articles from the above databases and articles were filtered based on whether they used transfer learning in breast ultrasound images or not. We excluded studies that did not involve ultrasound images though the studies were about breast cancer transfer learning as well as studies not written in English. Overall, 34 potentially relevant studies were selected, of which 30 remained after removing the duplicates in terms of methodology and dataset. Ten studies were rejected after screening their abstracts and titles. Based on our inclusion criteria, three reviewers assessed the full-length articles, and at this stage, 13 studies were excluded. Finally, we reviewed seven articles related to transfer learning in ultrasound breast images (Table 1). The experimental results from the reviewed papers demonstrate that the fine-tuned model exhibited the best performance. All of these studies applied cross-domain transfer learning, where the model trained on natural images is transfer learned to ultrasound images [23,24,25,26,27,28,29]. However, cross-modal transfer learning was implemented in [29], where a model trained on mammography images is transferred to breast MRI images, obtaining a better result. In all of the studies, transfer learning afforded better results than state of the art methods, because it uses a model that is pre-trained on a large dataset, such as ImageNet, with millions of image data, due to which it can predict new, unknown data. The most common pre-training models used in the transfer learning of breast ultrasound are the VGG19, VGG16, AlexNet, and InceptionV3 models (Table 1). ImageNet was used as a pre-training dataset in most breast ultrasound transfer learning. Different databases were utilized by the studies, including the open access series of breast ultrasound database (OASBUD) [26] with 200 ultrasound scans (two orthogonal scans each) of 52 malignant and 48 benign breast tumors; the UDIAT Diagnostic Centre of the Parc Tauli Corporation ultrasound image data (UDIAT) [24], which consists 163 ultrasound images corresponding to 110 benign and 53 malignant breast masses (one mass per image); ultrasound images obtained with BK Medical Panther 2002 and BK Medical Hawk 2102 (database A) [25,28], with 306 images, of which 246 are benign and 60 are malignant, and others acquired by researchers themselves, and mammograms as well as MRI datasets.

Transfer learning is utilized for different ultrasound imaging analyses purposes. A commonly used transfer learning approach is to pre-train a neural network on the source domain (e.g., ImageNet, which is an image database containing more than fourteen million annotated images with more than 20,000 categories and then fine-tune it based on the instances from the target domain (ultrasound). In [24], they used the VGG19 neural network, a CNN pre-trained on the ImageNet dataset that possesses five large blocks of convolutional layers and a fully connected layers block. They employed two transfer learning approaches. The first one employed the pre-trained model as a predetermined feature extractor where the model architecture was not modified. Second, they fine-tuned the CNN using the new dataset, breast ultrasound images. For the purpose of fine-tuning, the CNN structure was adjusted, the last layers of the architecture were replaced with different fully connected layers. Augmentation was applied on the main dataset to improve training, as well as to provide more mixed images to the network that increased the number of images six times more. In [25], the proposed transfer learning approach is based on fully convolutional networks (FCN-AlexNet) for semantic segmentation. FCN-AlexNet is a fully convolutional network version of the original AlexNet classification model with a few adjustments of the network layers for segmentation. In [26], in the case of the Inception V3 model, features for classification were extracted using the last average pooling layer. In the case of the VGG19 model, the first fully connected layer was used. In [27], VGG16, a 16-layer deep learning model that has been trained to classify images into 1000 categories was utilized. Models initialized with the VGG16 model do not need big numbers of labeled data or excessive computations. The parameters of the VGG16 model are used as an initialization of a fine-tuned model for the dataset under consideration. All of the convolutional layers were frozen except for the last one. Moreover, stochastic gradient descent (SGD) algorithm was employed in order to upgrade the network weights with the breast tumor dataset. Classifier training was carried on with 50 iterations. In [28], FCN-AlexNet, FCN version of the original AlexNet classification model with a few adjustments in the network layers for the segmentation task was used. The weights trained on ImageNet were transfer learned for semantic segmentation of breast ultrasound image with minor adjustments in the convolutionized fully connected layers. They initialized the weights of convolutional layers from the pre-trained models instead of using random weights. In [29], they employed fine-tuning using two CNN models: VGG-Net and MG-Net. VGG-Net is a very deep network that was originally trained with ImageNet data set. MG-Net was trained with the mammogram dataset for a similar mass detection task. The VGG-Net was VGG-128 that includes a fully connected layer with 128 outputs. The MG-Net architecture was made of three consecutive blocks, having nine convolutional layers.

Different performance analysis criteria (detection, classification, or segmentation) have been utilized in these previous studies on applying transfer learning to breast ultrasound.

A majority of detection methodologies employ seed point detection as an evaluation criterion [90,91]. In [90], a radiologist annotated a rectangular region of interest (ROI) with four extreme points, including the top, bottom, left, and right. Detection is treated as a true positive, provided that the detection apex (center of the segmented part) is located within the bounding box of the annotating expert radiologist. Otherwise, it is considered as a false positive (FP). In [25], a comparison of the performance of the detection methods in detecting breast cancer from ultrasound images was carried out using the true positive fraction (TPF) and false positives per image (FPs/image). The TPF is a measure of the sensitivity of the algorithm as in Equation (1). A few algorithms are capable of detecting several tumors, whereas others are only able to detect a single tumor. The TPF enables a smooth measurement because it quantifies the total number of detected tumors by taking the same amount equal to the total number of actual tumors [92,93,94]. Therefore, if a method can detect only one tumor in an image with multiple tumors, the TPF of this algorithm will be lower than the algorithm that has the ability to detect multiple tumors. In addition to the TPF and FPs/image as in Equation (2), another performance measure, referred to as the F-measure as in Equation (3) [95] (the weighted harmonic mean of recall and precision), is used to measure performance in detecting breast cancer from ultrasound imaging.

(1) TPF = (number of TPs)/(number of actual lesions)

(2) FPs/images = (number of FPs)/(number of images)

(3) F−measure = (2 × TP)/[(2 × TP) + FP + FN]

The area under the receiver operating characteristic (ROC) curve and AUC were calculated to evaluate breast cancer classification performance [96,97]. In [24], the AUC of the ROC curve was used to assess classification performance. The sensitivity, specificity, and accuracy of the classifiers were calculated based on the ROC curve, considering the point on the curve that was the closest to (0, 1).

The Dice similarity coefficient (Dice) was used to measure the accuracy of the segmentation results. Results of the Dice, sensitivity, precision, and Matthew correlation coefficient (MCC) were used as evaluation metrics in [27,28]. Table 2 summarizes the different performance analysis methods used.

It is easy to understand from Table 2 that different studies performance values differs. It is challenging to correctly describe the best transfer learning algorithm among the list in Table 2 because no study have been published to the best of our knowledge that compares the variety of transfer learning methods in ultrasound imaging except in [26] where a model pre-trained on Inception V3 outperformed VGG19 in the task of breast lesion classification. Furthermore, it can be observed from Table 2 that different works utilizing the same pre-training model (AlexNet in [23,25,29]) resulted in different performance value; this might be due to the different choices each work made during pre-processing and training.

## 4. Discussion

It is evident that transfer learning has been incorporated in various application areas of ultrasound imaging analyses [15,16]. Although transfer learning methods have constantly been improving the existing capabilities of machine learning in terms of different aspects for breast ultrasound analyses, there still exists room for improvement [84,85,86,87,88,89].

In [26], the results depict several issues related to neural transfer learning. First, the image reconstruction procedures implemented in medical scanners should be considered. It is important to understand how medical images are acquired and reconstructed [80,81,82,83]. However, there is limited information regarding the image reconstruction algorithms implemented in ultrasound scanners. Typically, researchers involved in computer-aided diagnoses (CADx) system development agree that a particular system might not perform well on data acquired at another medical center using different scanners and protocols [87]. Their study [26] clearly shows that this issue might also be related to the CADx system being developed using data recorded in the same medical center.

In [24], the authors presented that the lack of demographic variations in race and ethnicity in the training data can negatively influence the detection and survival outcomes for underrepresented patient groups. They recommended that future works should seek to create a deep learning architecture with pre-training data collected from different imaging modalities. This pre-trained model can be useful for devising new automated detection systems based on medical imaging.

In [27], the performance of fine-tuning is demonstrated to be better than that of the feature extracting algorithm utilizing directly extracted CNN features; the authors obtained higher AUC values for the main dataset. However, the implementation of the fine-tuning approach is by far challenging and difficult, relative to the feature extracting approach [24,25,26,27,28,29]. It requires replacement of the fully connected layers in the initial CNN with custom layers [84]. Additionally, identifying the layers of the initial model that should be trained in the course of fine-tuning is difficult [84]. Moreover, to obtain enhanced performance on the test data, the parameters must be optimally selected, and constructing a fine-tuning algorithm is time consuming [85]. Furthermore, with a small dataset, fine-tuning may not be advisable, and it would be wiser to address such cases using a feature extraction approach [75,76].

Therefore, several important research issues need to be addressed in the area of transfer learning for breast cancer diagnoses via ultrasound imaging. In [29], the authors hypothesized that learning methods pre-trained on natural images, such as the ImageNet database, are not suitable for breast cancer ultrasound images because these are gray-level, low-contrast, and texture-rich images. They examined the implementation of a cross-modal fine-tuning approach, in which they used networks that were pre-trained on mammography (X-ray) images to classify breast lesions in MRI images. They found that cross-modal transfer learning with mammography and breast MRI would be beneficial to enhance the breast cancer classification performance in the face of limited training data. This work can be used to improve breast ultrasound imaging by applying cross-modal transfer learning from a network pre-trained on mammography or other modalities.

The phenomenon of color conversion is extensively employed in ultrasound image analyses [27]. In [27], the authors showed that color distribution is an important constraint that should be considered when attempting to efficiently utilize transfer learning with pre-trained models. With the application of color conversion, it was proved that one could make use of the pre-trained CNN more efficiently [84,85,86]. By utilizing the matching layer (ML), they were able to obtain better classification performance. The ML developed was proved to perform the same when using other datasets as well [27]. Thoroughly studying these applications and improving the performance of transfer learning should be another potential research direction.

## 5. Outlook

It is customary that achieving better accuracy machine learning greatly depends on large training sample datasets. Nevertheless, compared with the available datasets in natural image domain, publicly available datasets in the field of medical ultrasound is still limited. The limited training data is a bottleneck for the further application of machine learning methods in medical ultrasound image analyses. To address the issue of small sample datasets, the commonly used method is transfer learning.

Effectiveness of a particular transfer learning algorithm for a given target task highly depends on two issues: source task as well as relation to the target. Generally, transfer learning would produce a sound learning between adequately related tasks while preventing negative transfer. However, achieving this is practically challenging. In order to overcome negative transfer, considering recognizing and rejecting detrimental source task knowledge, selecting the adequate source task from a set of potential source tasks, and designing the task similarity between multiple candidate sources tasks could be sound solutions. Furthermore, mapping is important for translating between task representations if the source and target tasks are different. Other challenges related to the use of transfer learning in machine learning include architecture selection, number of instances adequate to fine-tune besides the numbers of layers used in addition to the pre-trained model. Moreover, the effectiveness of transfer learning decreases when the target task (ultrasonic diagnosis) mismatches the source task (pre-trained network’s task).

Several directions are available for future research in the transfer learning area. First, transfer learning techniques can be further explored and applied to a wider range of applications in ultrasound image analysis. Second, how to measure the transferability across domains and avoid negative transfer is also an important issue. Although there have been some improvements on negative transfer, negative transfer still needs further systematic analyses. Third, the interpretability of transfer learning also needs to be investigated further. Finally, theoretical studies can be further conducted to provide theoretical support for the effectiveness and applicability of transfer learning. As a popular and promising area in machine learning, transfer learning shows some advantages over traditional machine learning, such as less data dependence and less label dependence in breast ultrasound imaging.

## 6. Conclusions

Transfer learning has facilitated the development of breast cancer diagnoses using ultrasound imaging, by overcoming the general challenge of obtaining a large set of training data using models that are pre-trained on a larger dataset of natural images, specifically, ImageNet. However, there remain issues that need to be addressed in order to achieve superior performance in terms of the pre-processing algorithms used and the dataset types for both the target and training tasks. Pre-processing techniques, such as color conversion, matching layer, and augmentation play a significant role in improving the performance of transfer learning; therefore, further research in this area should be the focus of future studies. Furthermore, most of the previous research on breast cancer diagnosis via ultrasound imaging has focused mainly on cross-domain transfer learning, although a few studies report the superiority of the cross-modal transfer learning method. Therefore, future studies should focus on applying cross-modal transfer learning and evaluate the performance under different applications of breast cancer diagnosis, including detection, classification, and segmentation.

## Figures and Tables

**Table 1:** Summary of previous transfer learning (TL) approaches for breast cancer diagnosis using ultrasound. OASBUD, open access series of breast ultrasound data; US, ultrasound; UDIAT, UDIAT Diagnostic Centre of the Parc Tauli Corporation ultrasound image data; dataset A, ultrasound images obtained with BK Medical Panther 2002 and BK Medical Hawk 2102; dataset B, UDIAT Diagnostic Centre of the Parc Tauli Corporation ultrasound image data.

Study	TL Approach Used	Pre-Training Model Used	Application	Image Dataset	Pre-Processing	Pre-Training Dataset
Byra et al. [26]	Fine-tuning	VGG19 & InceptionV3	Classification	OASBUD	Compression and augmentation	ImageNet
Byra et al. [24]	Fine-tuning	VGG19	Classification	882 US images of their own and public images UDIAT and OASBUD	Matching layer	ImageNet
Hijab et al. [27]	Fine-tuning	VGG16	Classification	1300 US Images	Augmentation	ImageNet
Yap et al. [25]	Fine-tuning	AlexNet	Detection	Dataset A and B	Splitting in to patches	ImageNet
Yap et al. [28]	Fine-tuning	AlexNet	Detection	Dataset A and B	Ground-truth labeling	ImageNet
Huynh et al. [23]	Feature extractor	AlexNet	Classification	Breast mammogram dataset with 2393 regions of interest (ROIs)	Compression and augmentation	ImageNet
Hadad et al. [29]	Fine-tuning	VGG128	Detection and classification	MRI data	Augmentation	Medical Image (Mammography image)

**Table 2:** Performance analysis criteria in transfer learning for diagnosing breast cancer from ultrasound images. AUC, area under curve; TPF, true positive fraction; FPs/image, false positives per image; F-measure, weighted harmonic mean of recall and precision; FCN, fully convolutional network; MCC, Matthew correlation coefficient; SVM, support vector machine; CNN, convolutional neural network.

Study	Performance Analysis Approach	Performance Metrics	Results
Byra et al. [26]	Classification performance of classifiers developed using train all and evaluated on test all.	AUC, sensitivity, accuracy, and specificity	InceptionV3: AUC = 0.857 VGG19: AUC = 0.822
Byra et al. [24]	Classification performance with and without the matching layer (ML) on two datasets. Bootstrap was used to calculate the parameter standard deviations.	AUC, sensitivity, accuracy, and specificity	The better-performing fine-tuning approach and matching layer, had AUC = 0.936 on a test data of 150 cases
Hijab et al. [27]	Comparison between accuracy of their model using ultrasound images and other related work.	AUC and accuracy	AUC = 0.98 Accuracy = 0.9739
Yap et al. [25]	Comparison of the capability of the proposed deep learning models on the combined dataset.	TPF, FPs/image, and F-measure	FCN-AlexNet (A + B): (TPF = 0.99 for A and TPF = 0.93 for B)
Yap et al. [28]	Dice similarity coefficient to compare with the malignant lesions.	Mean Dice, sensitivity, precision, and Matthew correlation coefficient (MCC)	“Mean Dice” score of 0.7626 with FCN-16s
Huynh et al. [23]	Classifiers trained on pre-trained models features were compared with classifiers trained with human-designed features.	AUC	SVM trained on human-designed features obtained an AUC = 0.90. SVM trained on CNN-extracted features obtained an AUC = 0.88
Hadad et al. [29]	Cross-modal and cross-domain transfers learning were compared.	Accuracy	Cross-modal = 0.93 Cross-domain = 0.90
